# Endemic shrubs in temperate arid and semiarid regions of northern China and their potentials for rangeland restoration

**DOI:** 10.1093/aobpla/plv063

**Published:** 2015-06-03

**Authors:** Jianmin Chu, Hongxiao Yang, Qi Lu, Xiaoyan Zhang

**Affiliations:** 1 Key Laboratory of Tree Breeding and Cultivation, State Forestry Administration; Research Institute of Forestry, Chinese Academy of Forestry, Beijing 100091, China; 2 Qingdao Engineering Research Center for Rural Environment, College of Resources and Environment, Qingdao Agricultural University, Chengyang, Qingdao, Shandong Province 266109, China; 3 Institute of Desertification Studies, Chinese Academy of Forestry, Beijing 100091, China

**Keywords:** Drought-enduring shrubs, endemic species, northern China, nurse plants, rangeland restoration, sand stabilization, species conservation, temperate arid regions

## Abstract

Some endemic shrubs in arid and semiarid ecosystems are in danger of extinction, and yet they can play useful roles in maintaining or restoring these ecosystems, thus practical efforts are needed to conserve them. The shrubs *Amygdalus pedunculata* Pall., *Amygdalus mongolica* (Maxim.) Ricker and *Ammopiptanthus mongolicus* (Maxim. ex Kom.) Cheng f. are endemic species in arid and semiarid regions of northern China, where rangeland desertification is pronounced due to chronic overgrazing. In this study, we tested the hypothesis that these endemic shrubs have developed adaptations to arid and semiarid environments and could play critical roles as nurse species to initiate the process of rangeland recovery. Based on careful vegetation surveys, we analysed the niches of these species in relation to precipitation, temperature and habitats. All sampling plots were categorized by these endemics and sorted by the non-metric multidimensional scaling method. Species ratios of each life form and species co-occurrence rates with the endemics were also evaluated. Annual average temperature and annual precipitation were found to be the key factors determining vegetation diversity and distributions. *Amygdalus pedunculata* prefers low hills and sandy land in temperate semiarid regions. *Amygdalus mongolica* prefers gravel deserts of temperate semiarid regions. *Ammopiptanthus mongolicus* prefers sandy land of temperate arid regions. Communities of *A. pedunculata* have the highest diversity and the largest ratios of long-lived grass species, whereas those of *A. mongolicus* have the lowest diversity but the largest ratios of shrub species. Communities of *A. mongolica* are a transition between the first two community types. These findings demonstrate that our focal endemic shrubs have evolved adaptations to arid and semiarid conditions, thus they can be nurse plants to stabilize sand ground for vegetation restoration. We suggest that land managers begin using these shrub species to restore degraded rangelands as part of a general conservation effort.

## Abstract (precis)

The shrubs *Amygdalus pedunculata*, *Amygdalus mongolica* and *Ammopiptanthus mongolicus* are endemic species in temperate northern China. They have developed adaptations to characteristic arid and semiarid ecosystems. *A. pedunculata* prefers low hills and sandy land in semiarid regions. *A. mongolica* prefers gravel deserts in semiarid regions. *A. mongolicus* prefers sandy land in arid regions. They play critical roles in maintaining or restoring these ecosystems. However, they are in danger of extinction. As part of a general conservation effort, they can be used as nurse plants to facilitate vegetation establishment in the engineering of rangeland restoration.

## Introduction

Rangeland desertification in semiarid and arid regions constitutes a global challenge for human welfare and social development (Dong *et al*. 2012; D'Odorico *et al*. 2013). With desertification, vegetation productivity and diversity are typically reduced, which further leads to serious disasters such as sand storms, sand drifts and sand burial (Kurosaki *et al*. 2011). Temperate northern China, with vast arid and semiarid rangeland, has experienced intensive desertification and associated vegetation and biodiversity loss for many years (Wang *et al*. 2013). Although major efforts have been launched to restore desertified rangeland, there is still disagreement about whether woody species or grasses are most appropriate for these restoration activities (Wang *et al*. 2008, 2010). Some scientists propose restoring the desertified land using grasses, rather than water-consuming woody species, while others worry that it will be difficult for grasses to become established on bare dunes without initial sand-stabilization provided by woody species, such as shrubs (Yang *et al*. 2006; Cao 2008; Padilla *et al*. 2009). The original vegetation of these regions should provide insights into this controversy, given that the resident plant species have so far survived the harsh environments of these regions and have presumably adapted to them (Wilson and Roberts 2011; Szitár *et al*. 2014).

The shrubs *Ammopiptanthus mongolicus* (Maxim. ex Kom.) Cheng f., *Amygdalus pedunculata* Pall. and *Amygdalus mongolica* (Maxim.) Ricker are endemic species, mainly distributed in arid and semiarid regions of northern China, and they have been officially designated as rare species deserving conservation efforts (Browicz and Zohary 1996; Liu 1998). These species now exist in remote locations that were never subject to severe overgrazing and desertification. They and associated plants constitute distinct communities of local original vegetation. As resident species, they may be promising candidates for rangeland restoration (Eldridge *et al*. 2011; Caperta *et al*. 2014; Kleinhesselink *et al*. 2014). We hypothesize that they have developed special adaptations to the conditions found in temperate arid and semiarid regions and can serve as nurse species to facilitate rangeland restoration. We therefore designed a study to investigate and analyse the diversity and structure of the original plant communities associated with these species, from which we wanted to collect factual evidence for the hypothesis.

## Methods

### Data collection

**Figure 1.**
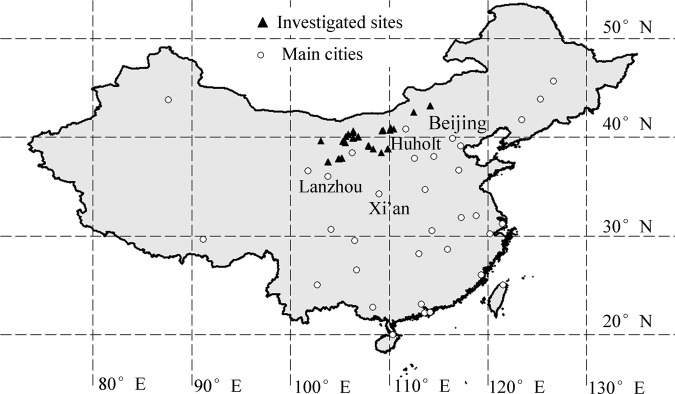
Study area and investigation sites in northern China.

The study area was located in arid and semiarid regions of northern China, where annual precipitation is mostly lower than 400 mm. Population patches of the three endemic shrub species were scattered in remote flatland and hills. During July and August of 2013, we selected 25 sites for field surveys, where nature reserves or protected areas have been established (Fig. 1). At each site, we randomly sampled three 10 × 10 m plots at intervals of ∼1 km. We measured higher shrubs (ca. >40 cm) individually, recorded their long and short crown diameters and calculated their crown area using the oval area formula. We classified these shrubs by species and calculated percent cover of each species, which was the total crown area of all shrubs of the same species divided by plot area. For other shrubs and grasses, we estimated percent cover of each species by counting their individual or patch numbers and measuring diameters of sample individuals or patches. All observed species were identified in the field by consulting handbooks of species identification (Lu *et al*. 2012). Because some traits were invisible or missing, we had to identify some plants at the genera level, belonging mainly to the families of Gramineae and Compositae. We also collected plot information such as longitude, latitude and elevation (using GPS, Garmin eTrex 30, Olathe, USA) and habitat type (i.e. sandy land, gravel land and rocky hills). Afterwards, we added the data of average annual precipitation (AnPre) and annual average temperature (AnTem) for every plot, by consulting published data for world climates (http://www.worldclim.org), covering the period from 1950 to 2000.

### Statistical analysis

We analysed niches of the three endemic shrub species in relation to AnTem and AnPre, using the HyperNiche 2 software (MjM Software, Gleneden Beach, OR, USA) (Carrick 2011). The data used for the analysis were percent cover of these species and values of AnTem and AnPre. The software explores the functional niche of a species through a series of parameter-flexible models. We chose the model type of quantitative local mean Gaussian and simulated with AnTem and AnPre concurrently. The software automatically performed iterative simulations and evaluated them by adjusted *R*^2^. We chose the simulations with the highest *R*^2^ as the best, where detailed significances (*p*) were not reported. We also conducted an Indicator Species Analysis using PCord 6.0 (MjM Software) to compare requirements of these shrub species for habitats (Peck 2010). A higher indicator value for a habitat type represents higher values of cover and frequency in habitats of this type. We did not simulate effects of the geographical factors of longitude, latitude and elevation, because they affect vegetation diversity and distribution indirectly and are too complicated to be well simulated.

The plots were sampled from an area spanning more than 1000 km, and their species composition was very heterogeneous. We found 184 species and 110 genera in these plots, many of which appeared only in one or two plots. We classified all observed species by families and calculated sum cover of every family in every plot, which was the sum of percent cover of all species of the same family. We sorted all the plots using PCord 6.0 with the non-metric multidimensional scaling (NMS) method, where vegetation data were the sum cover of every family and environmental data included AnPre, AnTem, longitude, latitude, elevation and habitat type (Peck 2010). The plots were categorized subjectively by the three endemic shrub-species.

All species were classified into three functional types according to life forms, i.e. short-lived grasses with longevity of 1 or 2 years, long-lived grasses with longevity >2 years and shrubs (including semi-shrubs) normally with longevity >2 years. We counted species number (richness) of every functional type in the plots. Based on species numbers, we calculated species ratios of each functional type in the plots; a species ratio of a functional type in a plot was species number of the functional type in the plot divided by total species number of all functional types in the plot. Then, we performed one-way ANOVA via Origin 8.0 (OriginLab, Northampton, MA, USA) to check whether species ratios of every functional type differed among the three plot-groups, which corresponded to the three endemic shrub-species. In addition, we evaluated how many species are inclined to co-occur with the three endemic shrub species by comparing their co-occurrence rates with these endemic shrub-species. The rates were times that a species co-occurred with an endemic shrub species divided by number of the plots where the pertaining endemic shrub-species occurred.

## Results

### Niches and habitats of the endemic shrub-species

**Figure 2.**
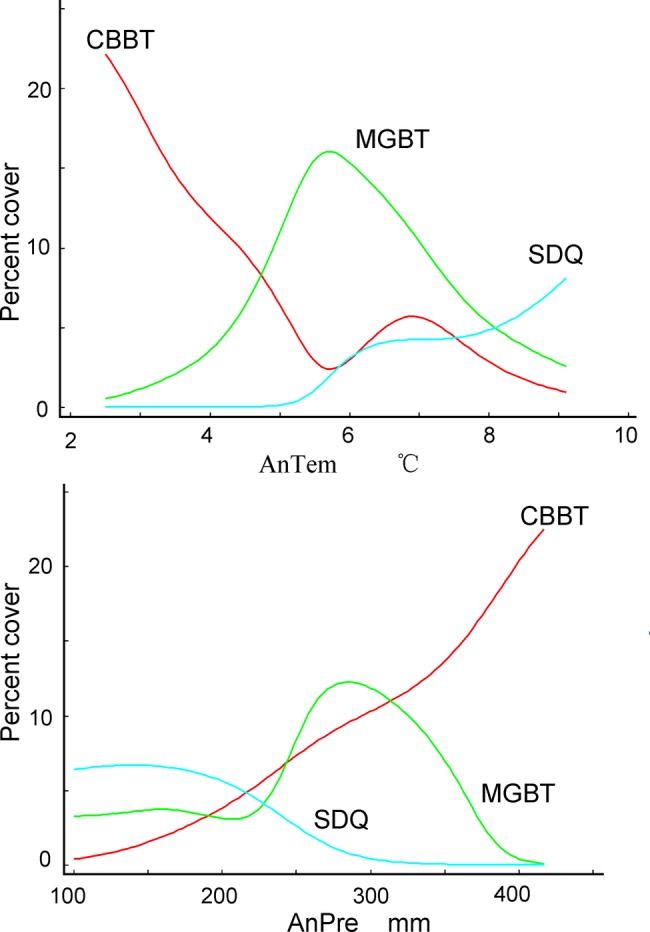
Functional niches and requirements of the endemic shrubspecies for climatic factors. Note: AnTem refers to annual average temperature and AnPre refers to average annual precipitation; CBBT is *A. pedunculata*, MGBT is *A. mongolica* and SDQ is *A. mongolicus*. The *R*^2^ of the simulations are 0.5006 for *A. pedunculata*, 0.5170 for *A. mongolica* and 0.4703 for *A. mongolicus*, where AnTem and AnPre are the two concurrent variables, although they are plotted in different sub-figures.

The best simulations with the highest *R*^2^ demonstrate niches of the three endemic shrub species for climates (Fig. 2). Optimal conditions for the species are discriminative. *Amygdalus pedunculata* grows best where annual average temperature is <4 °C and annual precipitation is more than 350 mm, with relatively higher cover than on other conditions. *Amygdalus mongolica* grows best where annual average temperature is 5.5 °C and annual precipitation is 280 mm. *Ammopiptanthus mongolicus* grows best where annual average temperature is more than 6 °C and annual precipitation is <200 mm. These shrub species are also discriminative in terms of habitat requirements. *Amygdalus pedunculata* prefers low hills and sandy land, *A. mongolica* prefers gravel deserts and *A. mongolicus* prefers sandy deserts (Table 1).

**Table 1.** Preferred habitats of the three endemic shrub-species in temperate arid and semiarid regions of northern China. *P < 0.05; **P < 0.01; ***P < 0.001.

Species	Indicator values			Significance (*P*)	Preferred habitats
	Hill	Gravel	Sand		
*A. pedunculata*	38	0	10	0.0034**	Low hills and sandy deserts
*A. mongolica*	7	48	7	0.0008***	Gravel deserts
*A. mongolicus*	3	0	41	0.0014**	Sandy deserts

### Main families and their distributions

**Figure 3.**
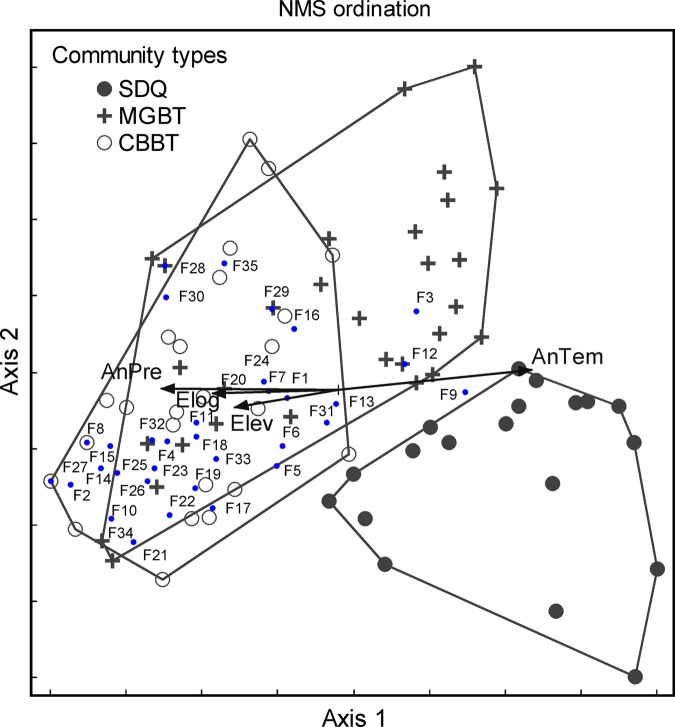
Non-metric multidimensional scaling ordination of sampling plots and plant families. Note: SDQ communities are characterized by *A. mongolicus*. CBBT communities are characterized by *A. pedunculata*. MGBT communities are characterized by *A. mongolica*. AnPre is average annual precipitation, AnTem is annual average temperature, Elong is east longitude and Elev is elevation or altitude. The factors of habitat type and north latitude were also used for the analysis, but they do not appear in the figure for inferior roles. The major families are Compositae (F11), Leguminosae (F6), Gramineae (F7), Rosaceae (F20), Chenopodiaceae (F12) and Zygophyllaceae (F9). The minor families include Liliaceae (F1), Ranunculaceae (F19), Labiatae (F4), Crassulaceae (F10), Plantaginaceae (F2), Tamaricaceae (F3), Polygonaceae (F13), Orobanchaceae (F14), Gentianaceae (F15), Asclepiadaceae (F16), Ephedraceae (F17), Verbenaceae (F18), Euphorbiaceae (F5), Saxifragaceae (F8), Thymelaeaceae (F21), Umbelliferae (F22), Cyperaceae (F23), Cruciferae (F24), Caryophyllaceae (F25), Rhamnaceae (F26), Berberidaceae (F27), Scrophulariaceae (F28), Convolvulaceae (F29), Ulmaceae (F30), Iridaceae (F31), Polygalaceae (F32), Rutaceae (F33), Boraginaceae (F34) and Bignoniaceae (F35).

The NMS ordination plot shows that AnTem and AnPre are the key factors determining vegetation structures and distributions, as indicated by their longer arrows (Fig. 3). In contrast, longitude and elevation play subordinate roles, perhaps because they are indirect factors to reduce or distort influences for intervening factors or processes. The ordination plot also reflects that: communities of *A. pedunculata* (CBBT, Chang-Bing-Bian-Tao in Chinese) tend to appear in relatively colder and wetter conditions, with higher plant diversity; those of *A. mongolicus* (SDQ, Sha-Dong-Qing in Chinese) tend to appear in warmer and drier conditions, with lower plant diversity and those of *A. mongolica* (MGBT, Meng-Gu-Bian-Tao in Chinese) often appear in transitional conditions between the communities of CBBT and SDQ, yet exhibiting higher similarity to CBBT communities and lower similarity to SDQ communities.

The plants are mainly from six families: Compositae (including 32 species, accounting for 17.5 % of the total species), Leguminosae (26 species, 14.2 %), Gramineae (26 species, 14.2 %), Rosaceae (14 species, 7.7 %), Chenopodiaceae (13 species, 7.1 %) and Zygophyllaceae (9 species, 4.9 %). The first four families are inclined to appear in plant communities of *A. pedunculata* or *A. mongolica*, namely, CBBT or MGBT communities. The latter two, Chenopodiaceae and Zygophyllaceae, tend to appear in plant communities of *A. mongolica* or *A. mongolicus*, namely, MGBT or SDQ communities. This indicates that SDQ communities are suitable for special plants despite a low diversity.

### Functional structure of plant communities

**Figure 4.**
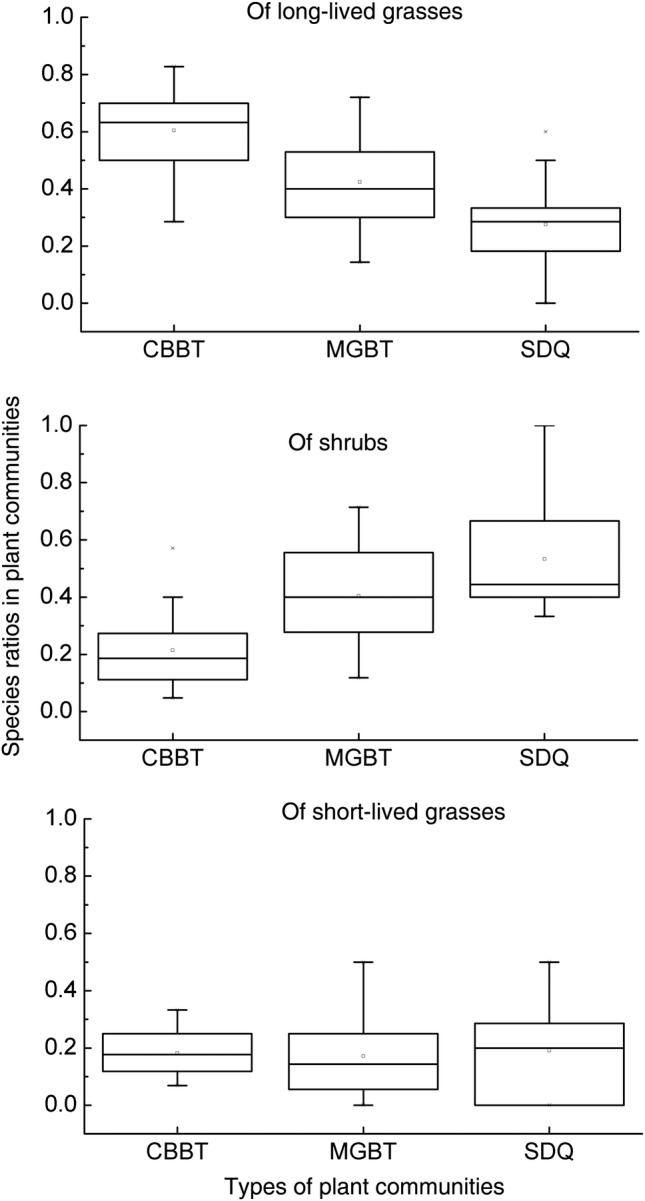
Species ratios of three life forms in different plant communities. Note: CBBT, MGBT and SDQ are community types characterized by *A. pedunculata*, *A. mongolica* and *A. mongolicus*, respectively.

The life form structures vary with community or plot types (Fig. 4). Species ratios of long-lived grasses are the highest in CBBT communities and the lowest in SDQ communities (*F*_2_,_72_ = 28.02, *P* < 0.001). Those of shrubs (including semi-shrubs) are the highest in SDQ communities and the lowest in CBBT communities (*F*_2_,_72_ = 20.20, *P* < 0.001). The features of MGBT communities are not as noticeable. This finding demonstrates that shrub species possess better potential to survive arid conditions than long-lived grasses. Especially, species ratios of short-lived grasses behave insensitively to arid and semiarid environments, i.e. show no significant differences among these community types (*F*_2_,_72_ = 0.14, *P* = 0.87).

### Species co-occurring with the endemic shrubs

The species often co-occurring with *A. pedunculata* included 17.4 ± 7.3 species: *Artemisia frigida*, *Stipa* spp., *Corispermum hyssopifolium*, *Cleistogenes squarrosa*, *Leymus secalinus*, *Potentilla acaulis*, *Artemisia ordosica*, *Psammochloa villosa*, *Heteropappus altaicus*, *Dracocephalum moldavica*, *Astragalus* spp., *Asparagus cochinchinensis*, *Artemisia sacrorum*, *Cynanchum thesioides*, *Ixeridium chinense*, *Allium mongolicum*, *Echinops sphaerocephalus*, *Caryopteris mongholica*, *Allium tenuissimum*, *Lespedeza daurica*. Those often co-occurring with *A. mongolica* included 12.5 ± 5.0 species: *Stipa* spp., *Allium mongolicum*, *Heteropappus altaicus*, *Reaumuria songarica*, *Pennisetum centrasiaticum*, *Zygophyllum xanthoxylum*, *Echinopilon divaricatum*. Those often co-occurring with *A. mongolicus* included 8.1 ± 4.7 species: *Zygophyllum xanthoxylum*, *Nitraria* spp., *Agriophyllum squarrosum*, *Allium mongolicum*, *Stipa* spp., *Cleistogenes squarrosa*, *Echinopilon divaricatum*. The listed species are those co-occurring at frequencies higher than 25 % with these endemic shrub-species. Others co-occurring at lower frequencies (<25 %) are omitted.

## Discussion

### Responses of shrubs and grasses to environments

The species ratios of shrubs (including semi-shrubs) increased from semiarid (CBBT communities) to arid conditions (SDQ communities), entirely opposite those of long-lived grasses and also unlike those of short-lived grasses. This reflects that native shrubs exceed grasses to endure arid conditions; that is, they have developed better adaptations to arid environments. For example, *A. pedunculata* grows well where annual precipitation is more than 350 mm, *A. mongolica* grows well where annual precipitation is ∼280 mm and *A. mongolicus* grows well where annual precipitation is <200 mm. Moreover, there are many other drought-enduring shrubs and semi-shrubs, such as *Nitraria* spp., *Zygophyllum xanthoxylum*, *Artemisia frigida*, *Artemisia ordosica* and *Reaumuria songarica*. In contrast, long-lived grasses are inclined to occur in semiarid conditions, presenting higher species ratios in semiarid CBBT communities than in arid SDQ communities. Plant diversity is found to be higher in semiarid communities, but is mainly attributed to long-lived grasses, rather than shrubs. Short-lived grasses do not show dependence on either arid or semiarid conditions, with approximate species ratios in SDQ, MGBT and CBBT communities.

The distinctive responses of plants in our system are likely explained with their life forms (Schulze *et al*. 1996; Throop *et al*. 2012; Venn *et al*. 2014). Grasses normally have most of their roots shallower than 20 cm, whereas shrubs have only part of their roots at such a shallow depth and allow many more roots to grow in soil deeper than 20 or 30 cm (Schulze *et al*. 1996; Yang *et al*. 2014). This difference determines their survival potentials. In semiarid regions, annual precipitation ranges from 200 to 400 mm, and in arid regions, it can be lower than 200 or 100 mm. With decreasing precipitation, sunny days increase and ground evaporation intensifies. Such evaporation takes place primarily in and near ground surface, and scarcely at depths >20 cm (Yuan *et al*. 2008). As a result, the surface soil from semiarid to arid regions turns increasingly drier, and accordingly becomes more intolerable for long-lived grasses. The situation is not so serious for many native shrubs because their roots can easily reach deeper than 20 or 30 cm, where snow melt and heavy showers are adequate to penetrate for storing and yet avoid ground evaporation (Berndtsson *et al*. 1996). Relying on such moisture storage, native shrubs can survive better than long-lived grasses, that is, stand vigorously to maintain an ecosystem (Schulze *et al*. 1996). As for short-lived grasses, because their roots are also shallow and their lives are transient, they usually act as wanderers among long-lived grasses and shrubs, seeking new spots for transient establishment (Bowers *et al*. 1995). Their occurrences depend mostly on uncertain chances, unlike long-lived grasses and shrubs, which compete for soil moisture at different depths through special roots and persistent endeavours. For this reason, species ratios of short-lived grasses remain approximate in different communities, seemingly independent of environmental aridity.

### Integration of rangeland restoration and species conservation

Now that native shrubs can exceed grasses to survive arid conditions, some of them may be qualified as nurse species to facilitate recovery of arid and semiarid ecosystems (Yang *et al*. 2006; Zhao *et al*. 2007; Kleinhesselink *et al*. 2014). After these ecosystems suffer serious desertification, their vegetation-free surfaces usually become sensitive to wind erosion and sand drifts (Li *et al*. 2004; Xu 2006). Plants have difficulty colonizing such bare ground, except for a few annuals that grow only on favourable days (Yang *et al*. 2006). Every spring, winds are particularly strong and ground surface is very dry, thus causing serious ground erosion, sand drifts and sand burial. In this case, grasses may be dug out and killed unless their cover is high enough to tightly stabilize ground sand (Yang *et al*. 2006). Relative to grasses, shrubs embed their roots much deeper, achieving more chances to survive. They therefore survive better than grasses by protecting most roots from exposure to the sun (Wu and Yang 2013). Sand burial is also common in arid areas, but is not as harmful as sand erosion, and may be favourable for seed germination and plant establishment (Shi *et al*. 2004). Branches and stems of pioneer shrubs can slow winds and stabilize sand, thereby facilitating sand and seed deposition around them (Yang *et al*. 2006; Zhang *et al*. 2013). This condition is also beneficial for seed germination and seedling growth (Shi *et al*. 2004; Zhao *et al*. 2007; Zhang *et al*. 2013). As a result, grasses and other shrubs can follow such pioneer shrubs to become increasingly established.

We suggest that the three endemic shrub-species, as native rarities already adapted to temperate arid and semiarid regions, are potential candidates for restoration projects in degraded rangeland systems (Zhao *et al*. 2007; Kleinhesselink *et al*. 2014). *Amygdalus pedunculata* is suitable for semiarid regions. *Amygdalus mongolica* is suitable for semiarid gravel regions. *Ammopiptanthus mongolicus* is suitable for arid sand regions. The seedling stage is a critical period for these species to become established because seedlings are highly sensitive to climatic events and damage by livestock (Padilla *et al*. 2011; Li *et al*. 2013). Once their seedlings get through this stage, they can grow to stabilize sand ground and facilitate subsequent establishment of more plants (Chillo *et al*. 2011; Le Stradic *et al*. 2014). In particular, as rare species deserving conservation, if they are chosen for rangeland restoration, conservation efforts for them are greatly advanced. On one hand, their growth area can be enlarged, so that they are no longer restricted to nature reserves and protected areas. On the other hand, people can benefit from these efforts, at least ensuring the process of rangeland recovery.

## Conclusions

The endemic shrubs of *A. pedunculata*, *A. mongolica* and *A. mongolicus* have developed adaptations to arid or semiarid environments. They are promising nurse species to initiate rangeland restoration, by facilitating sand stabilization and plant establishment. Because these endemics are also unique and precious, people may become enthusiastic to grow them in their degraded rangelands, as long as official encouragement is available.

## Sources of Funding

The study was supported by the National Natural Science Foundation of China (31370707, 31000322), the Basic Research Fund of CAF (CAFYBB2007043, 200714) and the Huimin Project of the Ministry of Science and Technology (2012GS610203).

## Contributions by the Authors

H.Y. and J.C. contributed equally to the study. H.Y., J.C. and Q.L. conceived the study; H.Y., J.C. and X.Z. performed research; H.Y. and J.C. analysed data and J.C. and H.Y. wrote the paper.

## Conflict of Interest Statement

None declared.
